# Improving the transitioning of pediatric patients with type 1 diabetes into adult care by initiating a dedicated single session transfer clinic

**DOI:** 10.1186/s40842-020-00099-z

**Published:** 2020-06-05

**Authors:** Sarah Williams, Leigh Anne Allwood Newhook, Heather Power, Rayzel Shulman, Sharon Smith, Roger Chafe

**Affiliations:** 1 Faculty of Medicine, Memorial University of Newfoundland and Labrador, St. John’s, Canada; 2 Janeway Pediatric Research Unit, Memorial University of Newfoundland and Labrador, St. John’s, Canada; 3 Division of Children and Women’s Health, Eastern Health, St. John’s, Canada; 4 Hospital for Sick Children, Toronto, Canada; 5 SickKids Research Institute, Toronto, Canada; 6 Institute for Clinical and Evaluative Sciences, Toronto, Canada

**Keywords:** Pediatric, Diabetes, Transition, Transfer clinic

## Abstract

**Background:**

Young adults with type 1 diabetes face potential health problems and disruptions in accessing care related to their move from pediatrics into adult care. At a medium-sized pediatric hospital with no formal transition support program, we developed and evaluated the use of a single-session transfer clinic as an initial quality improvement intervention to improve patient satisfaction, clinic attendance, and knowledge of transition related issues.

**Methods:**

Following a jurisdictional scan of other diabetes programs, the pediatric diabetes program developed a half-day transfer clinic. After the first transfer clinic was held, evaluation surveys were completed by patients, parents, and healthcare providers. Based on the feedback received, we altered the structure and evaluated the revised clinic by surveying patients and parents.

**Results:**

All patients and parents who attended reported high levels of satisfaction with the clinic. Providers were also mostly positive regarding their participation. Feedback from the first clinic was used to modify the structure of the second clinic to better meet the needs of participants and to allow the clinic to run more efficiently. The use of group sessions and adapting resources developed by other diabetes programs were viewed favourably by participants and lessened the burden on staff who delivered the clinic.

**Conclusions:**

A half-day transfer clinic is a viable step towards improving patient and parent satisfaction during the transition into adult care without requiring additional staff or significant expenditures of new resources. This type of clinic can also be incorporated into a larger program of transition supports or be adopted by programs serving young adults with other chronic diseases.

## Background

Young adults with type 1 diabetes face a range of potential healthcare issues as they move from pediatric care into adult care. Normal developmental issues during this period, such as living more independently for the first time, can be challenging for people who need to regularly monitor and manage their condition [1, 2]. Young adults with diabetes face increased difficulties in navigating the healthcare system, including often having minimal guidance on how the transition from pediatric to adult care will occur, longer waits to access care in the adult system, and adjusting to a new care environment and culture [1, 3]. Similarly, this transitional period represents a challenge for providers in retaining and providing appropriate care to their young adult patients [4]. All of these issues contribute to decreased clinic attendance, poorer treatment adherence and glycemic control, and increases in hospitalization rates and acute diabetes complications for young adults with diabetes [2, 5, 6].

The Canadian province of Newfoundland and Labrador (NL) has one of the highest reported incidence rates of type 1 diabetes globally, which from 2007 to 2010 was 49.9/100,000 (95% CI 42.2, 57.6) [7]. The Janeway Children’s Health and Rehabilitation Centre (often referred to as the Janeway) is the province’s only pediatric hospital. Until 2017, there were no dedicated transition supports, e.g., patient navigators, education booklets, local educational resources, available at the Janeway for young adults with diabetes as they prepared to move into the adult-focused healthcare system. There were also limited additional financial resources available within the diabetes program for initiatives to improve the care young adults received while transitioning out of pediatrics. Other clinical diabetes programs likely face similar circumstances.

## Methods

In this article, we detail the process the Janeway diabetes program used to develop, evaluate and revise a single session transfer clinic as an intervention to improve transition care. Describing this quality improvement process in detail will hopefully help other programs to develop similar clinics that are suited to their own level of resources and needs.

### Setting

The Janeway diabetes program cares for approximately 300 patients with type 1 diabetes, which represents over half of the provincial pediatric population with diabetes [8]. These patients are supported during their pediatric care by a multidisciplinary team consisting of pediatric endocrinologists, diabetes nurse educators, a nutritionist, a social worker, a schoolteacher, and a consultant clinical psychologist. Between 15 to 30 of its patients transition out of their pediatric care annually. The pediatric diabetes program does not assess transition readiness. Patients are transferred into adult care after they turn 18. The program does have some flexibility in retaining patients beyond their 18th birthday, e.g., young adults who are going on to post-secondary education usually are not transferred until they complete their first semester. Similarly, patient do not transfer until an appropriate provider is identified for them to transfer. At the time of this study, most patients transitioned from the Janeway to an adult-focused diabetes clinic located in another part of the city.

### The intervention: establishing the transfer clinic

In June 2016, the Janeway diabetes program held a retreat with local stakeholders, including adult-focused endocrinologists, to identify ways to improve pediatric diabetes care in NL. A key issue identified by stakeholders was the lack of preparation for young adult patients and their families as they transitioned into adult care. At that time, the transition support given to patients and families was neither structured nor uniform across providers, with issues being discussed, if at all, as part of regular clinic visits [8]. Structured transition programs have been shown to have a positive impact on the health outcomes of young adults with type 1 diabetes [9, 10], specifically with improved glycemic control and visit attendance as well as greater patient satisfaction [11, 12]. One recommendation from the retreat was to establish a structured transfer clinic as a first step for improving the transition experience for patients and their families.

A working group in the Janeway’s diabetes program was formed and began developing the clinic structure. The working group included three pediatric endocrinologists, a general pediatrician, two diabetes nurse educators, a dietician, a clinical psychologist, a social worker, and a research assistant. The working group first conducted a review of other diabetes transition programs, with a focus on programs in Canada. Through this review, the group identified educational resources which were adopted and revised to fit the local circumstances. These resources included a diabetes knowledge and skills checklist, a transition summary record form, and a booklet which overviewed issues for young adult patients with type 1 diabetes during their transition to adult care developed by Markham-Stouffville hospital (Ontario). Permission to use and adopt these resources was granted by their authors. The local adult diabetes team which accepted most of the Janeway’s patients after their transition also developed a small brochure introducing patients to their team.

### The first clinic

Patients in their last year of high school or first year of post-secondary education were identified and invited by the Janeway diabetes program staff to attend the first transfer clinic. All patients were between the age of 17 and 18. All patients were also using an insulin pump. Most of the patients with type 1 diabetes in the Janeway program would use an insulin pump as provincial health insurance provides universal coverage for pumps until patients reach 25 years of age.

Prior to the clinic, each participant was sent a letter explaining the aim of the clinic, a copy of the knowledge and skills checklist with an explanation that it was to be completed prior to the clinic, and the booklet which overviewed the issues for young adult patients with type 1 diabetes (Additional file 1). The introduction letter to patients also invited their parents to attend the clinic, as they were seen as key to successful transition. Patients were asked to download their insulin pump information as they would for a usual diabetes clinic visit. The diabetes team also discussed the transfer clinic with patients during their regular clinic visits months before to ensure that as many people as possible were familiar with the new clinic and its purpose.

The transfer clinic was intended to replace the patients’ final pediatric clinic visit and was designed to be completed in approximately 2 h (Table 1). The first transfer clinic was held in the same space in the pediatric hospital where patients usually attended their diabetes clinic visits and included sessions for both patients and their parents. During the transfer clinic, young adults met individually with a pediatric endocrinologist, a diabetes nurse, a dietician, and took part in a group session with a social worker. Each healthcare professional was given specific topics related to transition to discuss with the patients. The content covered included self-management of diabetes; troubleshooting insulin pump problems; prevention of hypoglycemia and diabetic ketoacidosis; staying well with diabetes; reducing the risk of complications; nutritional management of diabetes (topics such as carbohydrate counting and supermarket shopping tips); sexual health, alcohol and drugs; mental health issues and accessing medical assistance. A white board was used to track visits to ensure all patients were seen by each team member. At the same time, parents met in a group session – without their children - with the psychologist to discuss any concerns they had related to their child’s transition into adult care.

**Table 1:** Transfer clinic structure

First Transfer Clinic	Second Transfer Clinic
**Location:** Pediatric Diabetes Clinic	**Location:** Adult Diabetes Clinic
**Pre-Clinic:**	**Pre-Clinic:**
Introduction letter	Introduction letter
Knowledge and skills checklist	Knowledge and skills checklist
Booklet on issues for young adult patients with type 1 diabetes	Booklet on issues for young adult patients with type 1 diabetes
Download insulin pump information	Download insulin pump information
**Clinic Structure:**	**Clinic Structure:**
Patients had one-on-one sessions with the pediatric endocrinologist, diabetes nurse, and dietician; and one group session with a social worker	Patients had group sessions with a pediatric endocrinologist, diabetes nurse, dietician, and social worker
Parents had a group session with a psychologist	Parents had a group session with a psychologist
Received brochure introducing the adult diabetes team	Patients and parents met a member of the adult diabetes team
	Patients and parents toured the adult diabetes clinic space
	Received brochure introducing the adult diabetes team
**Post-Clinic:**	**Post-Clinic:**
Pediatric team completed the transition summary record form for each patient, which was then forwarded to the adult diabetes team	Pediatric team completed the transition summary record form for each patient, which was then forwarded to the adult diabetes team

All patients who attended the clinic were to be transitioned to a local adult diabetes program. This program is run by an adult endocrinologist and adult diabetologist. After the first transfer clinic, the pediatric team completed a transition summary record form for each patient who attended detailing status of their medical care. These medical summaries were then forwarded to the adult diabetes program. Medical summaries were not shared with the patients, although patients could request to see them as part of their medical record. The adult program then took over responsibility for contacting and booking patients for their next diabetes clinic visit.

Evaluation surveys were developed for patients, parents, and healthcare providers to ensure all relevant perspectives on the clinic were captured (Additional file 1). Evaluations were focused on how useful the clinic was, concerns about transferring out of pediatrics, the ability of the clinic to address these concerns, availability of resources to support patients during their transfer out of pediatric care, and suggestions of ways to improve the clinic. To protect confidentiality of participants, all surveys were anonymous and did not include patient identifiers. No clinic staff were present while participants completed the surveys.

## Results

The first transfer clinic was held February 7, 2017, with 6 young adults scheduled for the morning and 6 more for the afternoon sessions. Five patients did not attend or rescheduled, leaving 7 (58.3%) young adults with type 1 diabetes and 4 parents attending the clinic.

All patients and parents who attended the first clinic reported high levels of satisfaction (Table 2)*.* After the first clinic, 6 (85.7%) patients and 3 (75.0%) parents said that the clinic addressed concerns that they had about transition. All patients (100%) indicated that they would recommend this clinic to others with diabetes who are in the process of transferring out of pediatric care. After 3 months, the adult clinic reported that of the attendees at the first transition clinic, 6 out of 7 (85.7%) attended their first scheduled appointment and the remaining patient has an appointment pending. This illustrates that the transfer clinic can support the link into adult care.

**Table 2:** Summary of evaluation questions

	First Transfer Clinic				Second Transfer Clinic				Total			
	Patient *n* = 7		Parent *n* = 4		Patient *n* = 8		Parent *n* = 11		Patient n = 15		Parent *n* = 15	
	Number (%)		Number (%)		Number (%)		Number (%)		Number (%)		Number (%)	
	Yes	No	Yes	No	Yes	No	Yes	No	Yes	No	Yes	No
Was the clinic useful?	7 (100)	0	4 (100)	0	8 (100)	0	11 (100)	0	15 (100)	0	15 (100)	0
Did the clinic address concerns you had?^a^	6 (85.7)	0	3 (75.0)	1 (25.0)	6 (75.0)	2 (25.0)	11 (100)	0	12 (80.0)	2 (13.3)	14 (93.3)	1 (6.7)
Were there other topics you would have liked to discuss?	1 (14.3)	6 (85.7)	1 (25.0)	3 (75.0)	0	8 (100)	1 (9.1)	10 (90.9)	1 (6.7)	14 (93.3)	2 (13.3)	13 (86.7)
Do you think you will use the transition guide?	7 (100)	0			8 (100)	0			15 (100)	0		
Would you recommend this clinic to another person with diabetes who is about to transfer out of the Janeway?	7 (100)	0			8 (100)	0			15 (100)	0		

^a^One participant in the First Transfer Clinic did not respond to this question

### The second clinic

Based on the evaluation surveys and feedback given by staff to the research team, revisions were made to the clinic structure. A second transfer clinic was held on May 15th, 2017 with 8 patients and 11 parents attending. The clinic session was moved to the site of the adult diabetes program to which the patients will transition so that patients could become familiar with the new program’s location. All one-on-one meetings were replaced with group teaching, which both improved the flow of the session and decreased the repetitiveness of instruction for providers. Patients, without their parents, had group sessions with a pediatric endocrinologist, diabetes nurse, dietician, and social worker. Parents had a group session with a pediatric psychologist. Patients and parents then met members of the adult diabetes team and toured the adult diabetes clinic space together.

Another round of evaluation surveys were conducted after the second clinic. After the second transfer clinic, 6 (75.0%) young adults and 11 (100.0%) parents found that the clinic addressed most of their concerns. Parents suggested that starting the transition process a year earlier would be helpful. The majority of parents (90.9%) found that the second clinic was helpful, with one commenting that it “was beneficial to interact with other adults whose kids have type 1 diabetes.” Another participant said that they were “very pleased with the visit”. We did not evaluate attendance of their first appointment at the adult diabetes clinic for those patients who attended the second clinic.

## Discussion

Smaller pediatric diabetes programs can find it difficult to commit substantial resources to improving the transition care of their young adult patients. While still requiring some staff time to organize and run, we found that the single session transfer clinic allowed a pediatric diabetes program to improve the care that patients received, without requiring additional staff or clinical time. This quality improvement was partly achieved by leveraging resources that were developed at other centres and adopting a plan-do-study-act approach for the intervention [13]. We found that the transfer clinic had a positive impact on patient and parent experience and was strongly supported by both the pediatric and adult care staff.

The pediatric and adult diabetes programs continue to have transfer clinics. This transfer clinic is being once a year, with about 15 people attending on average. As we are committed to continuous improvement of these transfer clinics, we request from all participants to identify any suggestions for improving the program. Requests we received since our formal evaluation include more information on sexual health and information for lesbian, gay, bisexual, transgender, and queer patients, who may experience additional unique challenges with diabetes care [14]. Participants also felt issues related to mental health could be more thoroughly addressed, which is especially important because of the high risk for mental health issues in this population [15–18]. The clinic structure allows for successive clinics to be adapted, with suggestions from previous sessions being integrated in order to better prepare young adults for their transition. A similar clinic structure could also be developed to target emerging adults in these years before they transition as part of a wider program of transition care, which is in keeping with current age recommendations [19].

Menon et al. recently found that there is a need for some type of transition clinic for young adults with a chronic illness, including diabetes [20]. The question is how best to structure this type of clinic. Some have argued that telemedicine and shared medical appointments can be effective methods for engaging young adults [21], and there is some evidence for the effectiveness of telemedicine solutions [22]. Similarly, there is evidence for the use of transition coordinators and regular meetings with adult providers during the transition period [23]. One of the strengths of our article is that it describes a relatively low-resource, single session intervention which is not explored in depth elsewhere. It is an intervention that can easily be adapted to incorporate additional supports for young adults during their transition, like those outlined above. The descriptive elements of our article also illustrate how a medical program can proceed to improve and evaluate transition outcomes for their patients.

This study has several limitations. Our evaluation is of a single site with a relatively small patient population transitioning into adult care every year. While these conditions are likely also true for many small and medium sized pediatric diabetes programs, caution should be used in applying our results to other centers. Even though it was a small number of patients who completed the evaluation surveys, we still think that there is value in describing the process we used to develop the clinic, the clinic structure, and patients and providers reaction to it. While 100% of patients and parents who attended the clinic completed the evaluation, we were unable to test the clinic concept with patients who chose not to attend. These patients may be harder to reach and thereby may be at greater risk for having significant gaps in clinic attendance during the transition period. Unique types of interventions will likely be required to better engage this population and address any unique issues they may have. Participant surveys were anonymous, which limited our ability to conduct sub-group analysis or present more baseline demographic data. Finally, although we were able to evaluate patient and provider satisfaction with the clinic, it was felt that there was an insufficient number of patients to evaluate impacts on clinical outcomes, e.g., diabetes related hospitalizations, at this time. We recognize that further follow-up is needed. Our plan is to follow subsequent cohorts of patients who transitioned out of the clinic to determine differences between those patients who attended a transfer clinic and those who did not.

## Conclusions

Young adults with diabetes are at an increased risk for adverse events during their transition into adult care. Many of these risks can, however, be mitigated if proper supports are in place. While patient satisfaction with our new transition program was shown to be high, we have not yet examined the impact of the long-term use of this clinic on patients’ A1cs and other health outcomes such as longer-term clinic attendance. Nevertheless, this intervention is advantageous in that it requires a fairly minimal investment of staff time, it does not require any significant investment of new resources, it can be employed within small pediatric centres, and it can be easily incorporated into larger transition programs. Based on our overall experience, we would recommend a similar transfer clinic as a useful quality improvement invention for pediatric diabetes programs, or even programs with young adult patients with other chronic conditions, to try to better support their patients as they move into adult care.

## Supplementary information

**Additional file 1: Appendix A**. Recruitment Letter. **Appendix B**. Patient, Parent and Staff Evaluation Surveys

## Data Availability

All pertinent data analyzed during this study are included in this published article. Any further data or information not included in this article may be obtained from the corresponding author upon request.

## Abbreviations

CI: Confidence Interval
NL: Newfoundland and Labrador (Canada)
