# Supplementary material for: Improving the transitioning of pediatric patients with type 1 diabetes into adult care by initiating a dedicated single session transfer clinic
Source: Clin Diabetes Endocrinol. 2020 Jun 5;6:11. doi: 10.1186/s40842-020-00099-z (PMC7275548; doi:10.1186/s40842-020-00099-z)
Supplement: Supplementary file 1 — Additional file 1: Appendix A. Recruitment Letter. Appendix B. Patient, Parent and Staff Evaluation Surveys [file 40842_2020_99_MOESM1_ESM.docx]

**Appendix A:** Recruitment Letter

Dear [Patient’s Name]

As you are approaching 18 or, are already 18 years of age, the time has come to transition you to an adult diabetes provider. We are starting a new transition process at the Janeway. You will have a transition appointment as your last appointment with us on **February 7^th^, 2017**. This appointment will have a different format than your usual appointment. The aim of the appointment is to ready you for care in the adult system. We will spend approximately 1.5-2 hours reviewing diabetes education and management with a focus on issues commonly encountered by young adults. We want to ensure that you feel empowered to be in control of your diabetes and that we are sending you off with the best possible diabetes knowledge. As the focus of the appointment is on **your** skills and knowledge, you will be seen alone. During this time, your parents are welcome to attend a session with one of our team members to discuss the transition process from their perspective.

Please find a form attached where you can indicate any areas you would like to review**. Please have this filled out prior to attending your appointment.** It will help us to guide our discussion. Please also find a copy of a diabetes guide for young adults. You do not need to read this prior to your appointment, it is for you to keep as a resource. We will cover much of the information in the booklet during your appointment and will give you a printed copy of the booklet.

As transition appointments have a different structure from your usual appointments, we only offer them once a year. Please ensure you attend as **we will not be able to rebook** you for another transition appointment if you miss it.

We look forward to seeing you. As this is a new process which we are piloting, we will ask you a few questions at the end of the appointment to get your feedback. This will help us to improve things and make sure the transition appointment is helpful.

Sincerely,

**Appendix B:** Patient, Parent and Staff Evaluation Surveys

**Thank you for taking part in today’s Transition Clinic. We would like to know if you think this clinic was helpful, and if there are areas we can improve. Please take a few minutes to answer the following questions:**

Was today’s clinic useful to you? Yes ___ No ___

Comments: _________________________________________________________________________

____________________________________________________________________________________

____________________________________________________________________________________

What concerns do you have about transferring out of the Janeway to a new care team?

__________________________________________________________________________________________________________________________________________________________________________

_____________________________________________________________________________________

Did the clinic help you address these concerns? Yes___ No ___

Comments____________________________________________________________________________

__________________________________________________________________________________________________________________________________________________________________________

Were there other topics you would like to have discussed? Yes ___ No ___

If yes, please list _______________________________________________________________________

_____________________________________________________________________________________

_____________________________________________________________________________________

Do you think that you will use the transition guide? Yes ___ No ___

Do you think you would use it in a different form (e.g., App, website)? Yes ___ No ___

Which form? ___________________________________________________________

Are there other supports or resources you would like to be in place to help make transferring to a new care team easier for you? Yes ___ No ___

If yes, please list ______________________________________________________________________

____________________________________________________________________________________

____________________________________________________________________________________

____________________________________________________________________________________

Are there ways that you think we could improve the clinic? Yes___ No ___

If yes, please list _______________________________________________________________________

_____________________________________________________________________________________

_____________________________________________________________________________________

_____________________________________________________________________________________

Would you recommend this clinic to another person with diabetes who is about to transfer out of the Janeway? Yes ___ No ___

If no, please list reason __________________________________________________________________________________________________________________________________________________________________________

**Thank you for your participation!**

**Thank you for taking part in your child’s transition clinic appointment. We would like to know if you think this clinic was helpful, and if there are ways we can improve. Please take a few minutes to answer the following questions:**

What are your main concerns about your child transferring out of the Janeway?

_______________________________________________________________________________________________________________________________________________________________________________________________________________________________________________________________

Did your visit today help you address these concerns? Yes ____ No _____

Comments____________________________________________________________________________

_____________________________________________________________________________________

_____________________________________________________________________________________

What issues would you have liked to have been discussed further?

_______________________________________________________________________________________________________________________________________________________________________________________________________________________________________________________________

Do you think today’s visit was beneficial to you? Yes ____ No ____

Comments______________________________________________________________________________________________________________________________________________________________________________________________________________________________________________________

Did you feel that you were appropriately involved? Yes ___ No ___

Comments______________________________________________________________________________________________________________________________________________________________________________________________________________________________________________________

Are there ways that you think we could improve this visit for either you or your child? Yes ___ No ___

If yes, please list _______________________________________________________________________

__________________________________________________________________________________________________________________________________________________________________________

As a parent, are there other supports or resources you would like to be in place to help make transferring to a new care team easier for you? Yes ___ No ___

If yes, please list _______________________________________________________________________

__________________________________________________________________________________________________________________________________________________________________________

**Thank you for your participation!**

**Thank you for taking part in yesterday’s Diabetes Transition Clinic. In order to help evaluate this clinic, we would like to know if you think this clinic was helpful, and if there are areas we can improve. Please take a few minutes to answer the following questions:**

In your professional opinion, do you think this clinic was useful for the patient? Yes ___ No ___

Comments: _________________________________________________________________________

____________________________________________________________________________________

____________________________________________________________________________________

What general issues did patients, or their parents want to discuss with you? __________________________________________________________________________________________________________________________________________________________________________

_____________________________________________________________________________________

Did this clinic provide an adequate opportunity for you to address these concerns? Yes___ No ___

Comments ____________________________________________________________________________

__________________________________________________________________________________________________________________________________________________________________________

Were there other topics you would like to have discussed with the patient or their parents but did not have time for? Yes ___ No ___

If yes, please list _______________________________________________________________________

_____________________________________________________________________________________

_____________________________________________________________________________________

Are there other supports or resources you would like to see in place to help make transferring to a new care team easier for patients with type 1 diabetes or their parents? Yes ___ No ___

If yes, please list ______________________________________________________________________

____________________________________________________________________________________

____________________________________________________________________________________

____________________________________________________________________________________

How do you think we could improve the clinic?

_____________________________________________________________________________________

_____________________________________________________________________________________

_____________________________________________________________________________________

_____________________________________________________________________________________

_____________________________________________________________________________________

Do you think the clinic was a valuable use of your professional time? Yes ___ No ___

Please explain the reason for your answer.

________________________________________________________________________________________________________________________________________________________________________

_____________________________________________________________________________________

_____________________________________________________________________________________

Is there anything else you would like to say about yesterday’s clinic?

__________________________________________________________________________________________________________________________________________________________________________

_____________________________________________________________________________________

_____________________________________________________________________________________

_____________________________________________________________________________________

**Thank you for your participation!**
